# Using AnnoTree to Get More Assignments, Faster, in DIAMOND+MEGAN Microbiome Analysis

**DOI:** 10.1128/msystems.01408-21

**Published:** 2022-02-22

**Authors:** Anupam Gautam, Hendrik Felderhoff, Caner Bağci, Daniel H. Huson

**Affiliations:** a Institute for Bioinformatics and Medical Informatics, University of Tübingen, Tübingen, Germany; b International Max Planck Research School “From Molecules to Organisms,” Max Planck Institute for Biology Tübingen, Tübingen, Germany; c Cluster of Excellence: Controlling Microbes to Fight Infection, Tübingen, Germany; Cleveland Clinic

**Keywords:** microbiome analysis, taxonomy, functional analysis, alignment, protein sequences, NCBI-nr, AnnoTree, function, software

## Abstract

In microbiome analysis, one main approach is to align metagenomic sequencing reads against a protein reference database, such as NCBI-nr, and then to perform taxonomic and functional binning based on the alignments. This approach is embodied, for example, in the standard DIAMOND+MEGAN analysis pipeline, which first aligns reads against NCBI-nr using DIAMOND and then performs taxonomic and functional binning using MEGAN. Here, we propose the use of the AnnoTree protein database, rather than NCBI-nr, in such alignment-based analyses to determine the prokaryotic content of metagenomic samples. We demonstrate a 2-fold speedup over the usage of the prokaryotic part of NCBI-nr and increased assignment rates, in particular assigning twice as many reads to KEGG. In addition to binning to the NCBI taxonomy, MEGAN now also bins to the GTDB taxonomy.

**IMPORTANCE** The NCBI-nr database is not explicitly designed for the purpose of microbiome analysis, and its increasing size makes its unwieldy and computationally expensive for this purpose. The AnnoTree protein database is only one-quarter the size of the full NCBI-nr database and is explicitly designed for metagenomic analysis, so it should be supported by alignment-based pipelines.

## INTRODUCTION

Next-generation sequencing (NGS) has revolutionized many areas of biological research (1, 2), providing ever-more data at an ever-decreasing cost. One such area is microbiome research, the study of microbes in their theater of activity using metagenomic sequencing (3). Here, deep short-read sequencing, and improving performance of long-read sequencing, are helping to explore the roles and interactions of microbiomes in different environments.

The two initial tasks for any microbiome sequencing data set are (i) taxonomic analysis, that is, to determine which organisms are present in a microbiome sample, and (ii) functional analysis to determine which genes and pathways are present. Task i can be addressed, to a degree, using amplicon sequencing (targeting 16S rRNA, 18S rRNA, or internal transcribed sequencing, for example). However, a species- or strain-level taxonomic analysis, and task ii, both are best addressed using whole-genome shotgun sequencing, that is, metagenomics proper.

Metagenomic shotgun sequencing reads can be analyzed using a number of different approaches, such as alignment-free *k*-mer analysis (4–6), alignment against DNA references (7, 8), or translated alignment against protein references (9–13).

The DIAMOND+MEGAN approach uses DIAMOND (14) to perform translated alignment (short-read sequencing) or “frameshift-aware” translated alignment (long-read sequencing) of metagenomic reads or contigs against the NCBI-nr database (15). The resulting alignment files are then “meganized” or analyzed using MEGAN (16) so as to perform taxonomic and functional binning. A detailed protocol of this simple two-step pipeline is presented in reference 17.

During meganization, reads are assigned to taxonomic classes in the NCBI taxonomy (18) and to the GTDB taxonomy (19). Reads are also assigned to functional entities in EC (20), EggNOG (21), InterPro families (22), KEGG (23), and SEED (24, 25).

The DIAMOND+MEGAN pipeline was originally designed for use with the NCBI-nr database (10). The NCBI-nr database contains nonidentical protein sequences from GenBank CDS translations, PDB (26), Swiss-Prot (27), PIR, and PRF, covering all domains of life and viruses. In August 2021, NCBI-nr comprised over 420 million sequences, of which just over half, ≈213 million, belong to bacteria or archaea. The NCBI-nr database is not explicitly designed for use in metagenomic analysis, and its rapidly increasing size is making it unwieldy for this purpose.

To perform taxonomic binning, MEGAN assigns reads to the NCBI taxonomy, which contains more than 2.2 million nodes and is designed for “structuring communication concerning all forms of life on Earth” (18, 28). More recently, the GTDB taxonomy (19) was developed for the explicit purpose of taxonomic analysis of microbiome sequencing data. Here, we introduce support for the GTDB taxonomy in MEGAN. The DIAMOND+MEGAN pipeline now performs taxonomic binning of metagenomic reads according to both the NCBI taxonomy and the GTDB taxonomy.

AnnoTree (29) provides functional annotations from over 27,000 bacterial and 1,500 archaeal genomes obtained from the GTDB database (19). The total number of protein sequences contained in the AnnoTree database is 106,052,079. The AnnoTree database is approximately one-quarter the size of NCBI-nr and only half the size of the prokaryotic part of NCBI-nr. However, AnnoTree contains protein sequences from more metagenome assembled genomes (MAGs) than NCBI-nr, and these are more likely to be found in microbiome samples.

In this paper, we describe and provide the necessary files for performing DIAMOND+MEGAN analysis using the AnnoTree protein reference database, as an alternative to NCBI-nr, to analyze the prokaryotic content of metagenomic sequencing samples. To illustrate the utility of this approach, first we compare the performance of DIAMOND+MEGAN using NCBI-nr and AnnoTree protein reference sequences on a published mock community of 23 bacterial and 3 archaeal strains (30). Using 10 published data sets from a range of different environments and obtained using both short- and long-read sequencing techniques, we then demonstrate that AnnoTree-based analysis is twice as fast and has a higher assignment rate than NCBI-nr-based analysis when using the DIAMOND+MEGAN pipeline. We also compare examples of both the NCBI and GTDB taxonomic binning.

## RESULTS

Using the standard DIAMOND+MEGAN analysis pipeline (16), metagenomic sequencing reads are first aligned against the NCBI-nr database using DIAMOND, and then the resulting alignments are processed by MEGAN (or the command-line tool daa-meganizer) so as to perform taxonomic and functional binning. We will refer to this as an NCBI-nr run of the pipeline.

In this paper, we describe a new application of the DIAMOND+MEGAN pipeline in which DIAMOND alignment is performed against the AnnoTree protein database, and we refer to this as an AnnoTree run. To enable the pipeline to be run in this way, we provide (i) a FastA file containing all ≈106 million AnnoTree protein sequences and (ii) an SQLite database (https://www.sqlite.org) that contains mappings of AnnoTree protein accessions to taxonomic and functional classes in all supported classifications. In addition, we provide a set of Python scripts that can be used to update both the FastA file and the mapping database.

To allow a fair comparison between the two runs, when aligning against the NCBI-nr database, throughout this study, we only aligned against the prokaryotic entries of the NCBI-nr database.

### Performance on a mock community.

**FIG 1:**
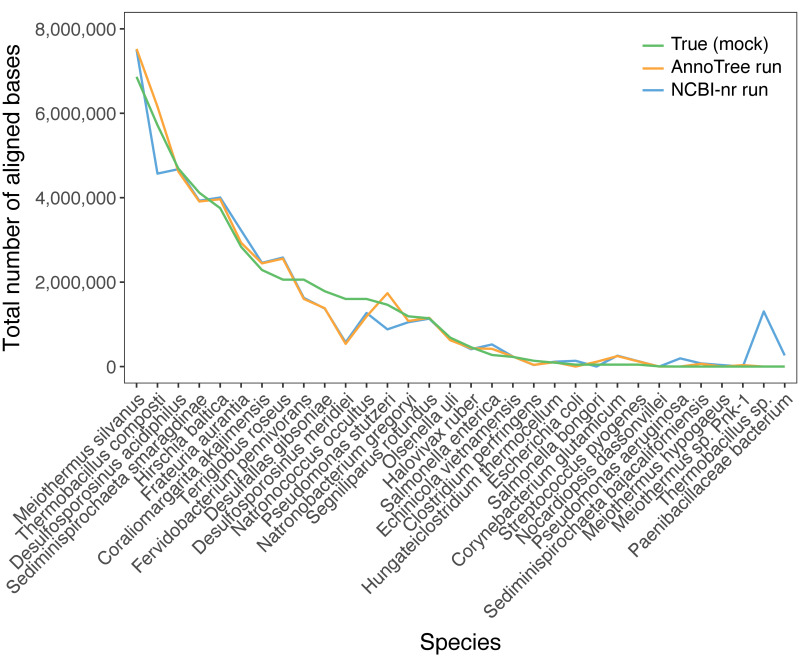
For prokaryotic species detected during analysis, we report the approximate relative abundance of organisms in the mock community (green) and the number of bases aligned and assigned by DIAMOND+MEGAN in an NCBI-nr run (blue) and in an AnnoTree run (orange).

For an initial comparison of the two ways of running the DIAMOND+MEGAN pipeline, we ran both on a set of 53,654 PacBio shotgun reads from the MBARC-26 mock community of 23 bacterial and 3 archaeal strains (30). In Fig. 1, we compare the two taxonomic profiles obtained from the NCBI-nr and AnnoTree runs with the published profile. Both computed profiles follow the published one quite closely, with some low-abundance false-negative assignments, namely, to Salmonella bongori and Escherichia coli for the former and latter run, respectively, and to Nocardiopsis dassonvillei for both. The NCBI-nr run gives rise to five false-positive bacterial assignments, namely, to *Paenibacillaceae bacterium*, Pseudomonas aeruginosa, *Sediminispirochaeta bajacaliforniensis*, Meiothermus hypogaeus, and *Thermobacillus* species. The latter has a large count that bleeds over from the high-abundance true-positive Thermobacillus composti. The AnnoTree run gives rise to two false-positive bacteria, *Sediminispirochaeta bajacaliforniensis* and *Meiothermus* sp. strain *Pnk-1*. There are no false positives or negatives in the archaea.

These results indicate that the use of the AnnoTree protein database, in place of the NCBI-nr protein database, is worth pursuing when the goal is to determine the prokaryotic content of a sample.

### Performance on 10 different data sets.

For a more detailed comparison of NCBI-nr and AnnoTree runs of the DIAMOND+MEGAN pipeline, we applied the two variants to a set of 10 different published samples that cover a range of different environments. Nine of the 10 data sets consist of short reads, and one consists of long reads. There are ≈580 million reads in total. The number of reads per data set is listed in Table 1. For each data set, we also report the number and percentage of reads that obtained a DIAMOND alignment against AnnoTree and NCBI-nr, respectively (Spearman’s correlation *ρ* = 1, *P = *2.2*e* – 16). In most cases, the ratio of the former number to the latter is ≈1, except in the case of the soil sample, where ≈50% more reads have an alignment against AnnoTree.

**TABLE 1:** Accession numbers and total number of reads for each data set^*a*^

Data set	Accession no.	Total no. of reads	Reads with DIAMOND alignments				Ratio
			AnnoTree (no.)	%	NCBI-nr (no.)	%	
River1	ERR466320	646,178	410,118	63.5	406,913	63.0	1.0
River2	SRR8859111	129,753,222	90,535,941	69.8	88,403,713	68.1	1.0
Seagrass	SRR6350025	98,260,754	36,053,215	36.7	33,717,202	34.3	1.1
Skin	ERR2538467	22,827,626	13,403,495	58.7	14,122,490	61.9	0.9
Stool	ERR2641811	33,214,614	29,132,562	87.7	30,101,313	90.6	1.0
Soil	SRR7521491	97,595,185	10,992,188	11.3	7,264,223	7.4	1.5
Thermal Pools	SRR6344961	52,908,626	15,751,382	29.8	16,625,446	31.4	0.9
Bioreactor1	SRR9831403	99,998,110	73,151,916	73.1	72,806,515	72.8	1.0
Bioreactor2	SRR8313048	44,258,996	36,608,649	82.7	37,477,641	84.7	1.0
Bioreactor3^*b*^	SRR8305972	694,827	613,958	88.4	616,536	88.7	1.0
Total		580,158,138	306,653,424	52.86	301,541,992	51.98	1.02

a For both the AnnoTree and NCBI-nr protein databases, we report the number and percentage of reads that obtained an alignment using DIAMOND. We also report the ratio between the two numbers.

b Long-read data set.

Out of the total of ≈580 million reads, DIAMOND aligns ≈307 million (≈52.9%) reads against the AnnoTree database and ≈302 million (≈52%) reads against the NCBI-nr database, that is, nearly 1% more reads against the former database, although the latter database contains more than twice as many entries.

### Taxonomic binning.

The DIAMOND+MEGAN pipeline assigns aligned reads both to the NCBI taxonomy (18) and, now, the GTDB taxonomy (19).

In the case of the NCBI taxonomy, performing AnnoTree runs on all data sets assigns 99.5% of all aligned reads to a taxonomic node, whereas the assignment rate when performing NCBI-nr runs is only 98.7% (Table 2). In total, using AnnoTree rather than NCBI-nr leads to the taxonomic assignment of  ≈7.6 million additional reads (≈1.3% of all reads). However, in more detail, the number of assigned reads is a few percentage points higher for the NCBI-nr runs on the two human-associated samples, skin and stool, and a few percentage points lower for the seagrass and soil samples.

**TABLE 2:** Assigned reads^*a*^

Classification	AnnoTree run			NCBI-nr run			Ratio
	Assigned	% of R	% of Al	Assigned	% of R	% of Al	
NCBI taxonomy	305,150,157	52.6	99.5	297,539,333	51.3	98.7	1.0
GTDB taxonomy	303,770,449	52.4	99.1	282,269,816	48.6	93.6	1.1
EC	78,874,545	13.6	25.7	76,552,285	13.2	25.4	1.0
eggNOG	95,932,149	16.5	31.3	87,131,284	15.0	28.9	1.1
InterPro	142,250,858	24.5	46.4	143,885,580	24.8	47.7	1.0
KEGG	209,371,499	36.1	68.3	123,130,673	21.2	40.8	1.7
SEED	102,452,692	17.7	33.4	100,615,086	17.3	33.4	1.0

a For each of the classifications provided by MEGAN and summarized over all AnnoTree runs and NCBI-nr runs of the DIAMOND+MEGAN pipeline on the 10 data sets listed in Table 1, we report the number of assigned reads (assigned), the percentage of all reads (% of R), and the percentage of all aligned reads (% of Al). In the last column, we report the ratio of the reads assigned using AnnoTree and NCBI-nr, respectively.

**FIG 2:**
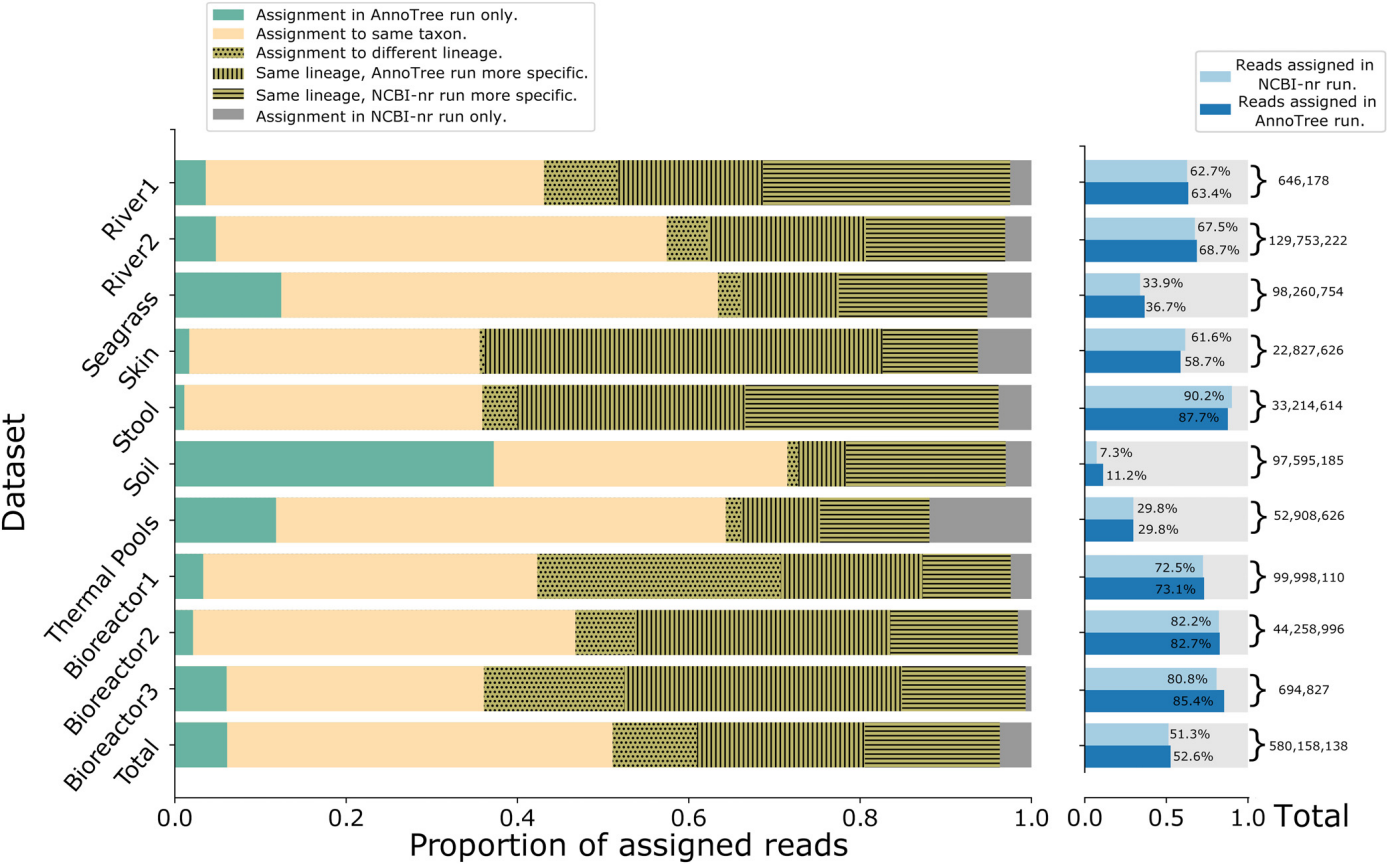
Details of the assignment of reads to the NCBI taxonomy. For each of the 10 data sets, for the total set of reads assigned by either an AnnoTree run or NCBI run of the DIAMOND+MEGAN pipeline, we show the proportion of reads only assigned by the AnnoTree run (green), assigned by both runs to the same taxon (yellow), or only assigned by the NCBI run (gray). For reads with differing assignments, we show the proportion assigned to incompatible lineages (dotted) or two compatible lineages with either the AnnoTree assignment being more specific (vertical stripes) or the NCBI-nr assignment being more specific (horizontal stripes). On the right, we indicate the total number of reads and the number of reads assigned by either the AnnoTree or NCBI-nr run.

For each of the 10 data sets, in Fig. 2 we present a more detailed comparison of the assignment of reads to the NCBI taxonomy for the AnnoTree and NCBI-nr runs. The results for both runs agree for 30 to 60% of all reads. For most data sets, the percentage of assigned reads that are assigned by only one of the two runs is similar, with the biggest exception being the soil sample, where approximately 40% of all assigned reads are assigned by AnnoTree only. For most data sets, the percentage of reads that are assigned incompatibly to different lineages is below or around 10%, with the exception of a bioreactor sample, where this value is around 30%.

In the case of the GTDB taxonomy, performing AnnoTree runs on all data sets assigns ≈99% of all aligned reads to a taxonomic node, whereas the assignment rate when performing NCBI-nr runs is only 93.6% (Table 2). In total, using AnnoTree rather than NCBI-nr leads to an assignment of ≈21.5 million additional reads (≈3.7% of all reads). On the stool sample, the assignment rate for the NCBI-nr run is 1% higher than that for the AnnoTree run, but in all other cases, the assignment rate for the AnnoTree runs is a couple of percentage points higher than that for the NCBI-nr runs.

**FIG 3:**
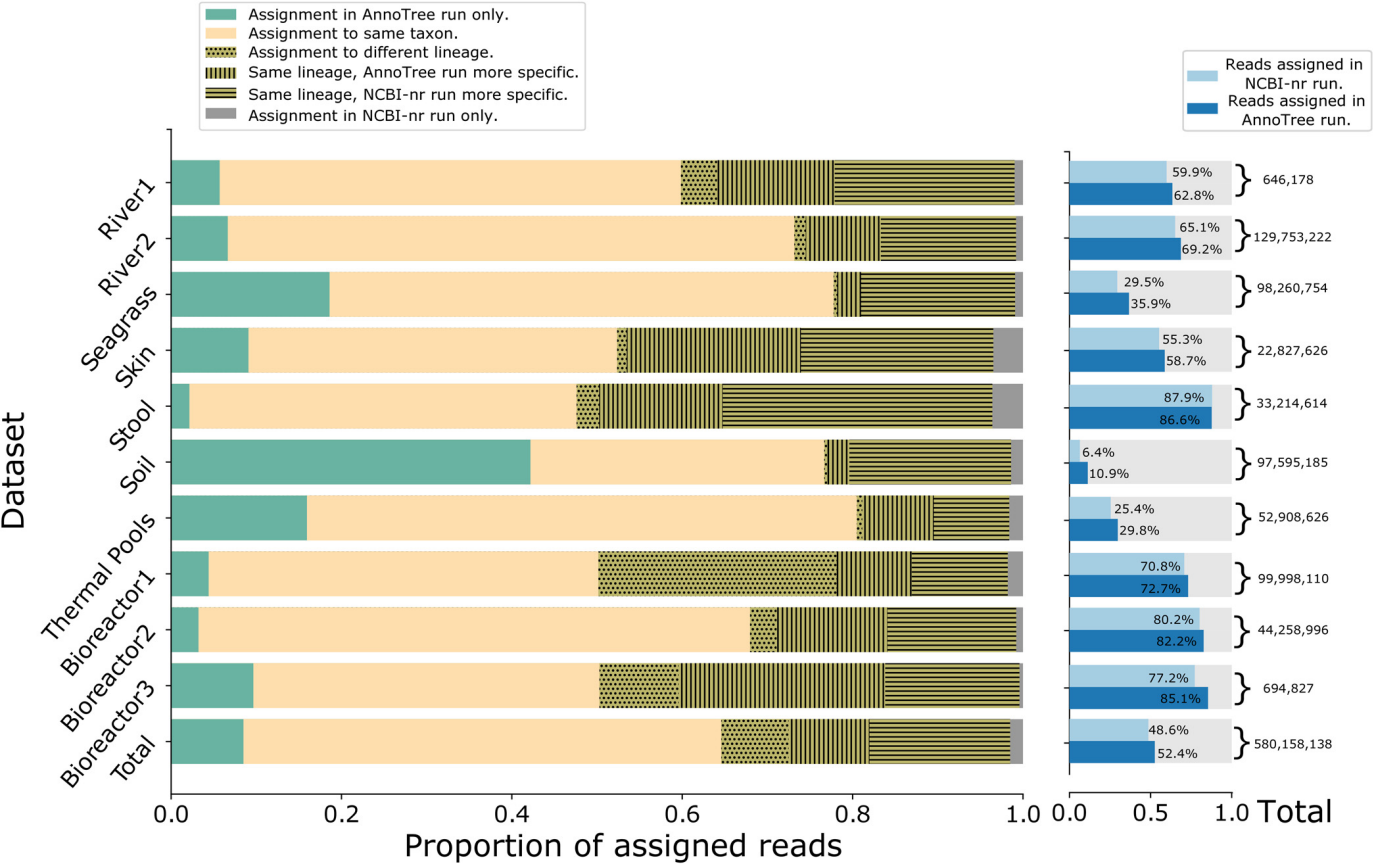
Details of the assignment of reads to the GTDB taxonomy, using the same colors as those in Fig. 2.

In Fig. 3, we present more details on the assignment of reads to the GTDB taxonomy. The results are similar to those for the assignment to the NCBI taxonomy but with a somewhat decreased level of conflicting assignments for most data sets.

### Functional binning.

The DIAMOND+MEGAN pipeline assigns aligned reads to a number of different functional classifications, currently EC numbers (20), eggNOG (21), InterPro families (22), KEGG (23), and SEED (24). For most functional classifications, the assignment rates for the AnnoTree runs are slightly higher than those for the NCBI-nr runs.

In the case of KEGG, performing AnnoTree runs on all data sets assigns ≈68.3% of all aligned reads to a KEGG node, whereas the assignment rate when performing NCBI-nr runs is only ≈40.8% (Table 2). AnnoTree runs make ≈70% more assignments of reads to KEGG nodes than the NCBI-nr runs do.

**FIG 4:**
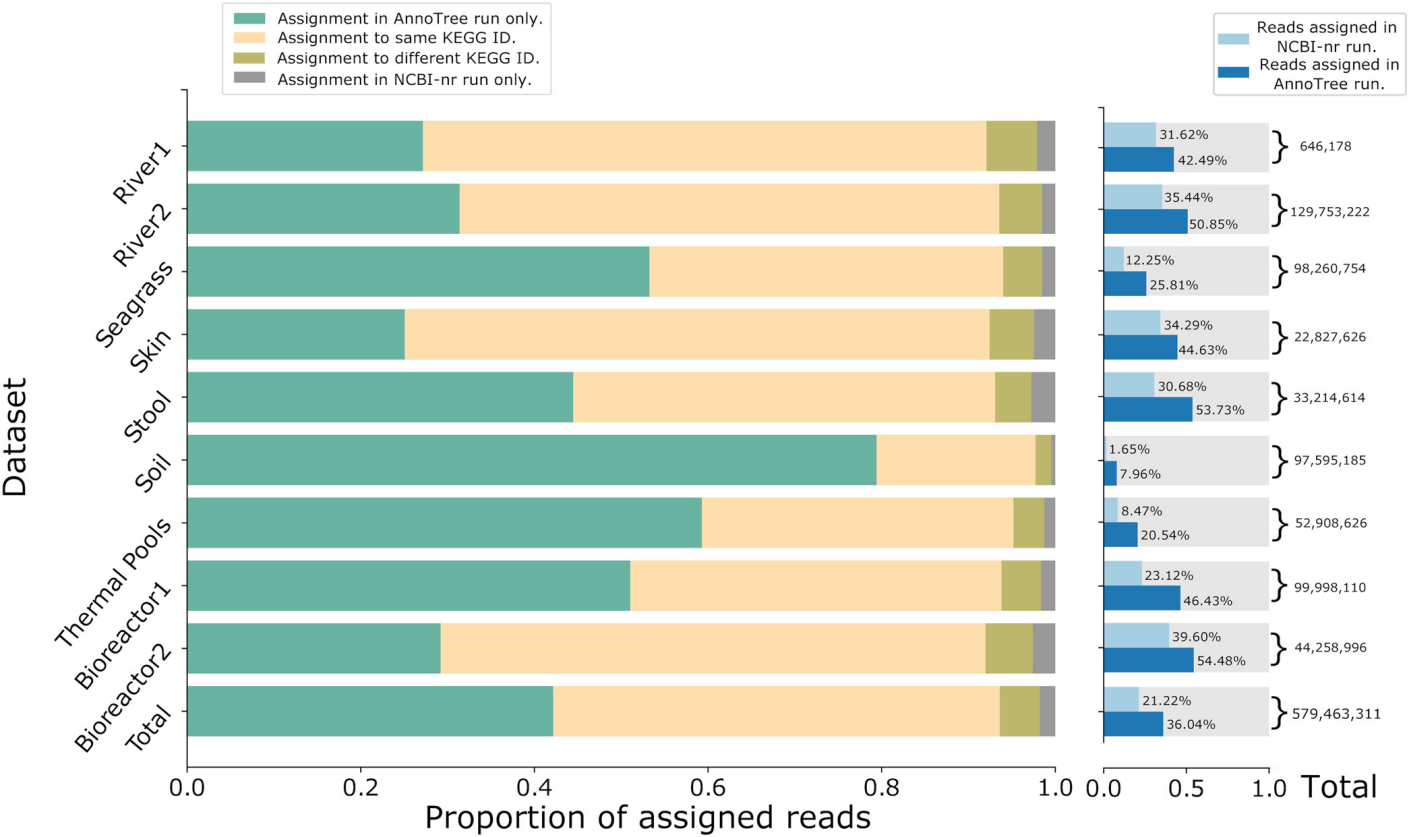
Details of the assignment of reads to KEGG. For each of the 10 data sets, for the total set of reads assigned by either an AnnoTree run or NCBI run of the DIAMOND+MEGAN pipeline, we show the proportion of reads only assigned by the AnnoTree run (green), assigned by both runs to the same class (yellow) or different classes (olive), or only assigned by the NCBI run (gray).

The number of read assignments to KEGG is particularly low for the soil sample, with AnnoTree-based assignment of only ≈8% and NCBI-based assignment of only ≈1.6% of all reads (Fig. 4).

### Running time.

As the AnnoTree protein database is less than one-quarter the size of the full NCBI-nr protein database, using the former during the alignment step of the DIAMOND+MEGAN pipeline will speed up the analysis. This will be offset, very slightly, by the fact that the number of aligned reads will be slightly higher. In the analysis performed here, we only aligned against the prokaryotic content of NCBI-nr, which contains approximately twice as many sequences as the AnnoTree protein database.

In Table 3, summarizing all 10 data sets, we report the CPU time used by DIAMOND, Meganizer, and both combined during both an NCBI-nr run and an AnnoTree run of the DIAMOND+MEGAN pipeline. On all data sets, DIAMOND alignment against the prokaryotic proteins in NCBI-nr takes about twice as long as alignment against the AnnoTree database, while meganization usually takes slightly longer in AnnoTree runs. In total, an AnnoTree run of the DIAMOND+MEGAN pipeline is twice as fast as an NCBI-nr run.

**TABLE 3:** CPU time used for running DIAMOND, Meganizer, and both combined^*a*^

DIAMOND			Meganizer			DIAMOND+MEGAN		
NCBI-nr	AnnoTree	Ratio	NCBI-nr	AnnoTree	Ratio	NCBI-nr	AnnoTree	Ratio
125,288 min	61,443 min	2.0	2,241 min	2,404 min	0.9	127,529 min	63,847 min	2.0

a Summarizing all 10 data sets, we show the CPU time used for running DIAMOND, Meganizer, and both combined during either an NCBI-nr run (restricted to prokaryotic sequences) or an AnnoTree run of the DIAMOND+MEGAN pipeline.

Performing an NCBI run using the full NCBI-nr database, not just the prokaryotic part, on each of the 10 data sets takes ≈3 times as long as an AnnoTree run, with an ≈4% increase of assignments to the NCBI taxonomy, on average.

## DISCUSSION

When first published in 2007 (10), MEGAN was run together with BLASTX (9) on data sets containing hundreds of thousands of short reads against the NCBI-nr protein database, which then contained around 3 million sequences. The DIAMOND alignment program (14) was later designed to allow the alignment of much larger data sets against a much larger NCBI-nr database. As the NCBI-nr database continues to grow, alignment against the full database presents an increasingly severe computational bottleneck.

As an alternative, projects focusing on the prokaryotic content of microbiome samples can make use of the GTDB taxonomy and the AnnoTree protein database, which are both explicitly designed for this. As the AnnoTree protein database is only 1/4 the size of the NCBI-nr database, DIAMOND alignment of reads against the AnnoTree protein database takes at most only half as much time while providing a superior assignment rate.

In this study, we illustrated the performance of AnnoTree runs using 10 example data sets from different environments. The results on these examples confirm that using AnnoTree is a useful alternative to using NCBI-nr.

The soil sample stands out. Its DIAMOND alignment rates against AnnoTree and NCBI-nr are very low at only 11.3% and 7.4%, respectively, in contrast to the alignment rates for the sool sample, which are very high at 87.7% and 90.6%, respectively. This illustrates that the diversity of the soil environment is only poorly represented in current databases (31, 32), while human stool samples and human-associated microbes have been studied in detail (33). The fact that the AnnoTree run has a higher assignment rate than the NCBI-nr run on the soil sample may be because the AnnoTree database recruits more sequences from metagenomic assembled genomes than NCBI-nr does.

To extend the AnnoTree-based approach to the detection and analysis of viral sequences, one could extend the AnnoTree protein database using either the virus subdivision of NCBI-nr or another dedicated resource (34, 35).

## MATERIALS AND METHODS

### Data sets.

We downloaded 53,654 PacBio shotgun reads (Sequence Read Archive [SRA] accession no. SRR3656744) from the MBARC-26 (Mock Bacteria ARchaea Community) data set (30), with a length of 11 to 16,403 and mean of 1,643.5. The true community profile reported in Fig. 1 was estimated from Fig. 2a of reference 36.

The 10 example data sets, listed in Table 4, were downloaded in FASTA format from the NCBI SRA using the NCBI SRA toolkit’s fastq-dump program: fastq-dump --split-spot --fasta 80 -I accession. Data sets with paired-end reads were concatenated into a single file. No additional preprocessing was performed.

**TABLE 4:** SRA run ID, sequencing platform, read layout, and total number of reads

Data set	SRA run ID	Platform	Layout	Total no. of reads
River1	ERR466320	LS454	Single	646,178
River2	SRR8859111	Illumina	Paired	129,753,222
Seagrass	SRR6350025	Illumina	Paired	98,260,754
Skin	ERR2538467	Illumina	Single	22,827,626
Stool	ERR2641811	Illumina	Paired	33,214,614
Soil	SRR7521491	Illumina	Paired	97,595,185
Thermal pools	SRR6344961	Illumina	Paired	52,908,626
Bioreactor1	SRR9831403	Illumina	Paired	99,998,110
Bioreactor2	SRR8313048	Illumina	Paired	44,258,996
Bioreactor3	SRR8305972	ONT	Single	694,827

In more detail, we used two data sets from rivers, River1 (https://www.ncbi.nlm.nih.gov/sra/?term=ERR466320) and River2 (37), one from the seagrass rhizosphere (38), one from the skin (39), one from the stool (40), one from the soil (41), one from thermal pools (42), and three from bioreactors (Bioreactor1 [43], Bioreactor2 [44], and Bioreactor3 [44]). Nine of the 10 data sets consist of short reads, whereas the last data set consists of ONT MinION long reads.

### Protein reference databases.

The NCBI-nr protein database was downloaded in January 2021 from the NCBI FTP site using the link ftp://ftp.ncbi.nlm.nih.gov/blast/db/FASTA/nr.gz. We also downloaded the two files prot.accession2taxid.gz (ftp://ftp.ncbi.nlm.nih.gov/pub/taxonomy/accession2taxid/prot.accession2taxid.gz) and nodes.dmp (ftp://ftp.ncbi.nlm.nih.gov/pub/taxonomy/taxdmp.zip).

A DIAMOND index was then generated using the following command (requiring 150 CPU minutes): diamond makedb --in nr.gz -d nr --taxonmap prot.accession2taxid.gz --taxonnodes nodes.dmp.

For both the AnnoTree Bacteria database and the AnnoTree Archaea database, we downloaded MySQL dump files (version of 25 August 2020) from the AnnoTree Bitbucket repository (https://bitbucket.org/doxeylabcrew/annotree-database/src/master/). These files were then imported into a MySQL server (version 5.7.35). The databases each have 21 tables, which hold information on the AnnoTree hierarchy, the GTDB taxonomy, the NCBI taxonomy, protein sequences, and additional mappings to Pfam, TIGRFAMs, and KEGG.

For each sequence in the “protein_sequences” table, we constructed a unique two-part accession string by concatenating its “gene_id” and “gtdb_id” values. For example, the protein sequence with gene identifier (ID) AE009439_1_1 and GTDB genome ID GB_GCA_000007185_1 was given the two-part accession AE009439_1_1__GB_GCA_000007185_1.

These accessions and the corresponding protein sequences (from both databases) were written to a FastA file, annotree.fasta, which we make available at https://software-ab.informatik.uni-tuebingen.de/download/megan-annotree.

A DIAMOND index was then generated using the following command (requiring 33 CPU minutes): diamond makedb --in annotree.fasta.gz -d annotree.

### MEGAN mapping databases.

We use the term “meganization” to refer to the process of analyzing the alignments of a set of sequences so as to perform taxonomic binning (for example, using the naive LCA algorithm for short reads or the interval-union LCA for long reads) and functional binning (usually using a best-hit approach). Meganization of DIAMOND alignments is performed either interactively using MEGAN or in a command-line fashion using the daa-meganizer program, which is bundled with MEGAN.

To perform meganization, MEGAN requires a so-called mapping database. This is an SQLite database file that contains a mapping of protein sequence accessions to all used taxonomical and functional classifications, namely, the NCBI taxonomy, the GTDB taxonomy, EC, EGGNOG, INTERPRO families, KEGG (MEGAN Ultimate Edition), and SEED. For NCBI-nr runs, we used the mapping database megan-map-Jul2020-2-ue.db, which we downloaded from https://software-ab.informatik.uni-tuebingen.de/download/megan6.

For AnnoTree runs, we created a new mapping database, called megan-mapping-annotree-June-2021.db, in SQLite format. This file is available at https://software-ab.informatik.uni-tuebingen.de/download/megan-annotree.

We used the above-described two-part accessions as the primary key for the mapping table. We determined the other entries of the mapping table as follows. The value for the GTDB and NCBI taxonomies were obtained from the “node_tax” tables of the two MySQL databases described above, using the gtdb_id part of the two-part accession.

The value for the KEGG classification was obtained from the “kegg_top_hits” tables of the two MySQL databases, using the gene_id part of the two-part accession. In the case that there is more than one possible KEGG assignment for a given protein, we randomly selected one. This was necessary because MEGAN allows at most one assignment per reference sequence. Both GTDB and KEGG IDs were additionally formatted to match the format required by MEGAN.

We calculated entries for the other classifications supported by MEGAN (EC, EGGNOG, INTERPRO, and SEED) by performing a join on the MD5 hash values of the protein sequences in the NCBI-nr and AnnoTree protein databases, in other words, by copying the classifications of an NCBI-nr accession over to an AnnoTree two-part accession whenever the two accessions correspond to the same protein sequence. We list the number of accessions that have assignments in the different classifications in Table 5.

**TABLE 5:** Number of prokaryotic accessions in NCBI-nr or AnnoTree that have map to a class in the classification systems^*a*^

Classification	NCBI-nr (no.)	AnnoTree (no.)	Ratio
NCBI taxonomy	182,329,414	106,052,079	1.72
GTDB taxonomy	126,956,422	106,052,079	1.2
EC	4,501,593	2,962,187	1.51
eggNOG	4,274,800	3,506,041	1.21
InterPro	19,748,423	11,069,757	1.78
KEGG	8,218,708^*b*^	56,577,432	0.15
SEED	31,117,272	16,183,436	1.92

a For two different taxonomical classifications (NCBI and GTDB) and for five different functional classifications (EC, EGG, InterPro, KEGG, and SEED) supported by MEGAN, we report the number of prokaryotic accessions in NCBI-nr or AnnoTree that have a mapping to a class in the classification and the corresponding ratio.

b MEGAN ultimate edition.

For most functional classifications, the number of AnnoTree proteins with assignments is smaller than that for NCBI-nr proteins, which is because the AnnoTree assignments are copied from NCBI-nr assignments. In the case of KEGG, the assignments are obtained from the AnnoTree database, which appears to maintain a much richer mapping than previously provided by MEGAN.

### Data set processing.

We ran DIAMOND (v2.0.4.142) on each short-read data set as diamond blastx -d 〈index〉 -q 〈input〉 -o 〈output〉 -f 100 -b24 -c1. Here, 〈index〉 is the index file, either annotree or nr, computed as described above. The input file, in (compressed) FastA or FastQ format, is specified by 〈input〉, and the output file is specified by 〈output〉. We used -f 100 to specify output in DAA format. The two remaining options, -b24 -c1, were used in an attempt to tune performance.

In addition, for purposes of comparison against the AnnoTree database, when running DIAMOND on NCBI-nr, we used the option -taxonlist 2,2157 to restrict alignment to bacteria (taxon ID 2) and archaea (taxon ID 2157).

When processing long reads, we also specified the -long-reads option.

The resulting DAA files were meganized using the daa-meganizer program MEGAN (version 6.21.5, ultimate edition, built 5 May 2021) as tools/daa-meganizer -i 〈input〉 -mdb 〈mapping〉. Here, the input file 〈input〉 is a DAA file produced by DIAMOND, and the mapping file 〈mapping〉 was either megan-map-Jul2020-2-ue.db or megan-mapping-annotree-June-2021.db, depending on whether the DIAMOND run was against the NCBI-nr or AnnTree protein database, respectively. When processing long reads, we also specified the -lg option.

### Data comparison.

We used the MEGAN tool daa2info to extract the mapping of reads to taxonomic and functional classes obtained in both the NCBI-nr and AnnoTree runs of the DIAMOND+MEGAN pipeline.

The following command was used to extract the mapping of reads to classes for all classifications: tools/daa2info -i 〈input〉 -o 〈output〉 -l -m -r2c Taxonomy GTDB KEGG EC EGGNOG INTERPRO2GO SEED. Here, the input file 〈input〉 is a meganized DAA file and the output file 〈output〉 is a text file. The output was used to determine the assignment rates for different classifications.

Similarly, the following command was used to extract the mapping of reads to taxonomic paths in the NCBI taxonomy: tools/daa2info -i 〈input〉 -o 〈output〉 -l -m -r2c Taxonomy -p true -r true. The output was used to generate Fig. 2.

The following command was used to extract the mapping of reads to taxonomic paths in the GTDB taxonomy: tools/daa2info -i 〈input〉 -o 〈output〉 -l -m -r2c GTDB -p true -r true. The output was used to generate Fig. 3.

### Computational resources.

The DIAMOND+MEGAN pipeline was run on a Linux virtual machine (provided to us by de.NBI Cloud, highmem xlarge) with Ubuntu 18.04.4 LTS operating system, Intel Xeon Gold 6140 CPU, 2.30 GHz (processor model name), 28 sockets, 1 core per socket, 1 thread per core, 28 CPUs (on-line CPU list: 0 to 27), 504 GB of RAM, and 6 TB of hard disk space. Reported run times are “user time” as calculated using the Linux “time” command. Furthermore, for MEGAN, the RAM size was set to 500 GB. All other calculations were undertaken on a MacBookPro laptop with a 2.6-GHz 6-core (12 threads) Intel Core i7 processor and 16 GB 2400-MHz DDR4 RAM.

### Statistical analysis.

Spearman’s correlations were computed using ggplot2 (45).

### Data availability.

All data sets analyzed here are publicly available from NCBI SRA, using the accession numbers listed in Table 4. The AnnoTree protein FastA file and mapping database both can be downloaded from https://software-ab.informatik.uni-tuebingen.de/download/megan-annotree.
